# Exposure to tobacco impressions during prime-time TV among Chilean minors by sex and socioeconomic status

**DOI:** 10.18332/tid/155264

**Published:** 2022-11-09

**Authors:** Armando Peruga, Carla Castillo-Laborde, Isabel Matute, Xaviera Molina, Oscar Urrejola, Ximena Aguilera

**Affiliations:** 1 Facultad de Medicina Clínica Alemana, Universidad del Desarrollo, Concepción, Chile; 2 The Bellvitge Biomedical Research Institute (IDIBELL), Barcelona, Spain; 3 Center for Biomedical Research in Respiratory Diseases (CIBERES), Instituto de Salud Carlos III, Barcelona, Spain

**Keywords:** minors, SES, Chile, tobacco imagery, TV

## Abstract

**INTRODUCTION:**

We tested if tobacco impressions were delivered differentially to prime-time TV watching minors by sex and socioeconomic status.

**METHODS:**

Programs aired during prime-time for three random weeks in 2019 from the 15 highest audience channels in Chile were content-analyzed for the occurrence of tobacco for each one-minute interval of 92639 recorded. Such occurrences were categorized as actual use and whether they violated Chilean smoke-free law or tobacco brand appearances. We estimated the number of persons per hour (p/h) exposed to tobacco impressions for the 4 to 17 years age group by sex and socioeconomic status (SES).

**RESULTS:**

Minors spent over a billion p/h watching TV during the observation period. Minors were exposed to tobacco explicit use, branding and smoke-free violation impressions for 9.7 million, 1.2 million, and 1.0 million p/h, respectively. The odds ratios (OR) of exposure to total tobacco impressions were always greater among boys with higher SES compared to boys with low SES. However, they were greater among girls of low SES compared to those of high SES for all types of impressions. The OR of exposure to tobacco branding was higher among girls of any SES compared to boys of any SES.

**CONCLUSIONS:**

Minors need protection from tobacco imagery on television, particularly girls of low SES. To that end, new legislation should implement all measures to counter depictions of tobacco in entertainment media, as recommended in the WHO FCTC Article 13 guidelines. This should require strong anti-tobacco advertisements before any TV program portraying tobacco targeting minor audiences, particularly girls of low SES. Given that Chile has one of the highest prevalences in the world of current cigarette smoking among young females, the potential contribution of tobacco impressions on TV to smoking differentials across female socioeconomic groups should be further studied.

## INTRODUCTION

Exposure to tobacco imagery in movies has been associated with smoking initiation, reinforcing tobacco use among smokers, especially adolescents and young adults, and facilitating the relapse of ex-smokers^1,2^. In Chile, feature films and animated productions were the main sources of exposure to tobacco impressions on TV for young people. Prime-time TV programming delivered millions of impressions of actual tobacco use to young people in 2019, at a rate of 8000 impressions per hour of TV viewing^3^. Broadcast TV programs may present or represent a tobacco product or its consumption. Such content is termed a tobacco occurrence. When the occurrence is delivered to and watched by an actual audience, we call it a tobacco impression.

Given that smoking prevalence in Chile is amongst the highest in the world among adults^4^ and females aged 13–15 years^5^ and considering that such smoking prevalence is not distributed equally across socioeconomic groups^6^, we tested if tobacco imagery on TV was delivered differentially to minors by sex and socioeconomic status, potentially contributing to influencing smoking across these groups. In this study, more specifically, we compare the tobacco impressions received by viewers of prime TV, aged <18 years, by sex and socioeconomic status in Chile.

## METHODS

The methodology of the study – sample frame, coding procedure and reliability, and outcomes – has been previously described^3^. In summary, we analyzed the tobacco occurrences on the prime-time TV content of the 15 most-watched channels in Chile for three weeks of 2019. Tobacco occurrences were classified as actual use and whether they violated Chile’s smoke-free law or the appearance of tobacco brands. Each of the existing 92639 one-minute intervals was classified as either 0 or 1, depending on whether a tobacco occurrence was observed.

In this study, we present the population reach of TV tobacco content for the audience aged 4–17 years. Reach is the population exposure per unit time to tobacco imagery and was calculated by occurrence type, multiplying the number of tobacco occurrences in each 1-minute interval by the estimated average number of people watching at that moment and summing them across all 15 TV channels. It is expressed as persons/hour (p/h) of viewership by sex and socioeconomic status (SES). Live audience viewing figures were collected and provided by Kantar IBOPE Media Chile from their peoples’ meter study (https://www.kantaribopemedia.cl/).

The peoples’ meter study classifies households according to a standardized socioeconomic index (ISE-YEO according to the acronym in Spanish) elaborated by the Chilean Association of Market Researchers. The ISE-YEO is a composite index of: a) the total household capita income according to household size adjusted according to household economies of scale, b) the highest formal education achieved by the main breadwinner in the household, and c) the occupation of the main breadwinner in the household^7^. The households are classified into 7 categories (A, B, C1, C2, C3, D and E) relative to what is considered the poverty line which receives an ISE-YEO of 1.00. Group E has an ISE-YEO value of below 1.00 and includes households considered under extreme poverty. The rest of the groups from D to A, have 5% incremental values of the ISE-YEO with groups A and B having values at least 25% above the poverty line. For this study, we have classified the households into two SES groups: 0–10% above the poverty line value, and ≥11% than the poverty line value. For ease of reference, we name these two groups low SES (groups C2–D) and high SES (groups A–C1). In 2019, 13% of Chilean households were considered living in extreme poverty, 62% were considered of low SES, and 25% of higher SES^8^. In Greater Santiago, perhaps better representing the urban Chile captured in the peoples’ meter study, the household distribution was as follows: 7% under extreme poverty, 58% in low SES and 35% in higher SES6. The peoples’ meter study of TV audiences does not include households in group E, and in addition only includes households in the urban spaces of the most populated areas of the country which include about 40% of the total population of Chile. The peoples’ meter study, therefore, targets a distribution of the households by socioeconomic group similar to that of the Greater Santiago.

Using multivariable weighted logistic regression, we assessed the odds of exposure to tobacco impressions. We estimated the odds ratio (OR) of exposure to each type of tobacco occurrence for each of the four sex and SES groups and compared them to the exposure in the females of the higher SES group. ORs were considered statistically significant for p<0.05. The analysis was conducted using STATA V.13.

## RESULTS

Table 1 describes the number of p/h exposed to three different types of tobacco impressions by sex and SES. A total of 1128.9 million p/h in this age group watched TV during the observation period. Of these, 9.7 million p/h, 1.2 million p/h, and 1.0 million p/h were exposed to explicit use, tobacco branding and smoke-free violation impressions, respectively. In other words, 0.87%, 0.11% and 0.22% of the persons/hour of people aged 4–17 years watched TV containing explicit use of tobacco, tobacco branding and smoke-free violation impressions, respectively.

**Table 1:** Persons per hour (p/h) of minors aged 4–17 years exposed to tobacco impressions, by type of impression

*Type of impression*			*Exposed to impressions*				
	*Sex*	*Socioeconomic status*	*No (Million p/h)*	*Yes (Million p/h)*	*Percent exposed*	*OR (95% CI)*	*p*
**Explicit use**	Male	Low	490.7	4.1	0.82	1.05 (1.04–1.06)	<0.0001
		High	121.2	1.1	0.92	1.17 (1.16–1.19)	<0.0001
	Female	Low	392.0	3.6	0.90	1.15 (1.14–1.16)	<0.0001
		High (Ref.)	115.3	0.9	0.79	1	
	Both	All	1119.2	9.7	0.87		
**Tobacco branding**	Male	Low	494.5	0.3	0.06	0.60 (0.59–0.62)	<0.0001
		High	122.3	0.1	0.08	0.84 (0.81–0.87)	<0.0001
	Female	Low	394.9	0.7	0.17	1.80 (1.76–1.85)	<0.0001
		High (Ref.)	116.1	0.1	0.10	1	
	Both	All	1127.8	1.2	0.11		
**Smoke-free violation**	Male	Low	493.7	1.0	0.20	1.48 (1.44–1.51)	<0.0001
		High	122.1	0.3	0.23	1.69 (1.64–1.73)	<0.0001
	Female	Low	394.6	1.0	0.25	1.84 (1.80–1.88)	<0.0001
		High (Ref.)	116.0	0.2	0.14	1	
	Both	All	1126.4	2.5	0.22		

Table 1 also describes the OR of being exposed to the different types of tobacco impressions by sex and SES compared to girls of the high SES group. In those aged 4–17 years, the odds of exposure were always greater among males of higher SES than among males of low SES compared both to females of high SES and among females of low SES compared to females of high SES for all types of impressions. However, the ORs were lower among females of higher SES than in the other sex/SES groups for all types of impressions, except for branding images. Exposure to branding impressions was notably higher for females in the low SES compared to those in the higher SES group. Nevertheless, such exposure was smaller for males in the low and higher SES compared to females in the higher SES group.

## DISCUSSION

Exposure to tobacco impressions was a small percentage (<1%) of the total persons/hour of TV watching in those aged 4–17 years. However, this represented millions of impressions received at the population level. Our results also show that exposure to tobacco impressions is higher among females of low SES compared to those with higher SES. Interestingly, SES seems to have a reverse exposure effect among males. Those of higher SES were always more exposed to tobacco impressions.

The study does not allow us to ascertain whether the differences in exposure by sex and SES translate into higher chances of initiating smoking of the different sex and SES subgroups. However, there are two facts that suggest that our results could impact an increased smoking prevalence, particularly among young Chilean girls.

First, the relationship between low SES and smoking has been well described. There is a robust association between the higher prevalence of current smoking and lower income levels. This association is particularly stronger among females^9^. In Chile, the existing data indicate that the smoking prevalence is higher among people of lower socioeconomic status^6,10^.

Second, numerous observational and a few experimental studies have documented an association between exposure to on-screen smoking and adolescent smoking^11^. One study also points to the SES level of adolescents as a mediating factor in smoking initiation^12^. Therefore, the fact that, in 2019, Chile had one of the highest prevalence in the world of current cigarette smoking among females aged 13–15 years (26.4%)^5^ could be related to the level of exposure to on-screen smoking seen in our study. However, other factors may mediate the role of low SES adolescents since these also experience higher exposure to other smoking risk factors such as family smoking, peer smoking, and poor school performance^13^.

### Limitations

The results of our study are limited in that they underestimate the population reach of tobacco impressions because the audience data available from the Kantar Ibope Peoples’ Meter survey extrapolates audience data to approximately only 40% of the Chilean population. Also, although the main source of watching TV for minors continued to be broadcast TV, our analysis does not include the exposure to tobacco imagery on streaming platforms, the internet and other entertainment venues, which are increasingly watched in the age group analyzed.

## CONCLUSIONS

Our research points to the need to protect minors from tobacco imagery on television, particularly girls of low SES. To that end, new legislation should implement all measures to counter depictions of tobacco in entertainment media recommended in the WHO FCTC Article 13 guidelines. This should require strong anti-tobacco advertisements prior to any TV program portraying tobacco targeted at audiences of minors, particularly girls of low SES. Finally, given that Chile has one of the highest prevalences in the world of current cigarette smoking among females aged 13–15 years, our results are of interest to further ascertain the potential contribution of tobacco impressions on TV to smoking differentials across socioeconomic groups among females.

## Data Availability

The data supporting this research are available from the authors on reasonable request.
